# Use of a novel drape ‘tent’ as an infection prevention control measure for mastoid surgery

**DOI:** 10.1017/S0022215120002637

**Published:** 2020-12-02

**Authors:** R J Lawrence, G M O'Donoghue, P Kitterick, D E H Hartley

**Affiliations:** 1 National Institute for Health Research Nottingham Biomedical Research Centre, UK; 2 Hearing Sciences, Division of Clinical Neuroscience, School of Medicine, University of Nottingham, UK; 3 Department of Otolaryngology, Nottingham University Hospitals NHS Trust, UK

**Keywords:** COVID-19, Coronavirus, Otolaryngology, Mastoidectomy, Otology

## Abstract

**Background:**

Mastoid surgery is an aerosol-generating procedure that involves the use of a high-speed drill, which produces a mixture of water, bone, blood and tissue that may contain the viable coronavirus disease 2019 pathogen. This potentially puts the surgeon and other operating theatre personnel at risk of acquiring the severe acute respiratory syndrome coronavirus-2 from contact with droplets or aerosols. The use of an additional drape designed to limit the spread of droplets and aerosols has been described; such drapes include the ‘Southampton Tent’ and ‘OtoTent’.

**Objectives:**

To evaluate the use of a novel drape ‘tent’ that has advantages over established ‘tent’ designs in terms of having: (1) a CE marking; (2) no requirement for modification during assembly; and (3) no obstruction to the surgical visual field.

**Results and conclusion:**

During mastoid surgery, the dispersion of macroscopic droplets and other particulate matter was confined within the novel drape ‘tent’. Use of this drape ‘tent’ had no adverse effects upon the surgeon's manual dexterity or efficiency, the view of the surgical field, or the sterility. Hence, our findings support its use during mastoid surgery in the coronavirus disease 2019 era.

## Introduction

As we pass through the first peak of the coronavirus disease 2019 (Covid-19) pandemic, healthcare specialist bodies are striving to resume non-Covid-19 related services on the premise of a safe working environment for all staff.^1^ However, the use of high-speed drills is known to be associated with the generation of droplets^2,3^ and aerosols,^4^ and for this reason mastoid surgery is considered an aerosol-generating procedure.^5–7^

A droplet is defined as a very small drop of fluid (10–100 μm) that travels over short distances. An aerosol can travel over longer distances, and refers to a suspension of fine solid or liquid droplet in a gas, with a diameter of 10 μm or less.^8–10^ Coronavirus disease 2019 is known to spread either through direct contact with an object that has the virus on it, or through indirect contact via the inhalation of droplets from an infected person.^11^ Although Asadi *et al*. suggested that transmission of the Covid-19 pathogen (severe acute respiratory syndrome coronavirus-2 (SARS-CoV-2 virus)) may also occur via aerosols,^12^ this remains controversial.^11^

It is highly feasible that the middle ear of a Covid-19 positive patient contains SARS-CoV-2,^13^ given its connection with the viral-laden nasopharynx^14^ via the Eustachian tube. However, despite other coronaviruses having been found in the middle ear,^15^ there have been no reported investigations of SARS-CoV-2 in this anatomical location to date. Regardless, mastoid surgery is considered to be high risk for the transmission of Covid-19 from an infected patient to healthcare workers via the drill-induced dispersion of a mixture of water, bone, blood and tissue.^6^

Infection prevention control measures that aim to reduce aerosolisation and contain the spread of droplets and other matter during mastoid surgery have been described. These include the use of an additional or multiple drape(s) to create a ‘tent’ over the surgical field, such as the ‘Southampton Tent’,^6^ the ‘Ototent’^16^ and other methods.^17,18^ Recent pre-clinical studies have suggested that the use of such a drape ‘tent’ could reduce the dispersion of particulate matter during temporal bone drilling.^16,17,19^ However, all of these established and previously evaluated ‘tent’ designs require modifications during the assembly process, or are associated with a significant amount of ‘spare’ drape that can interfere with surgical efficiency.^6,18,19^

We evaluated the use of a novel drape ‘tent’ that has advantages over other designs in terms of having: (1) a CE marking; (2) no requirement for modification during assembly; and (3) no significant ‘spare’ drape gathering around the surgical field.

## Evaluation of novel drape ‘tent’

The evaluation of the novel drape ‘tent’ occurred during a mastoid surgical procedure performed on a two-year-old child.

**Fig. 1.**
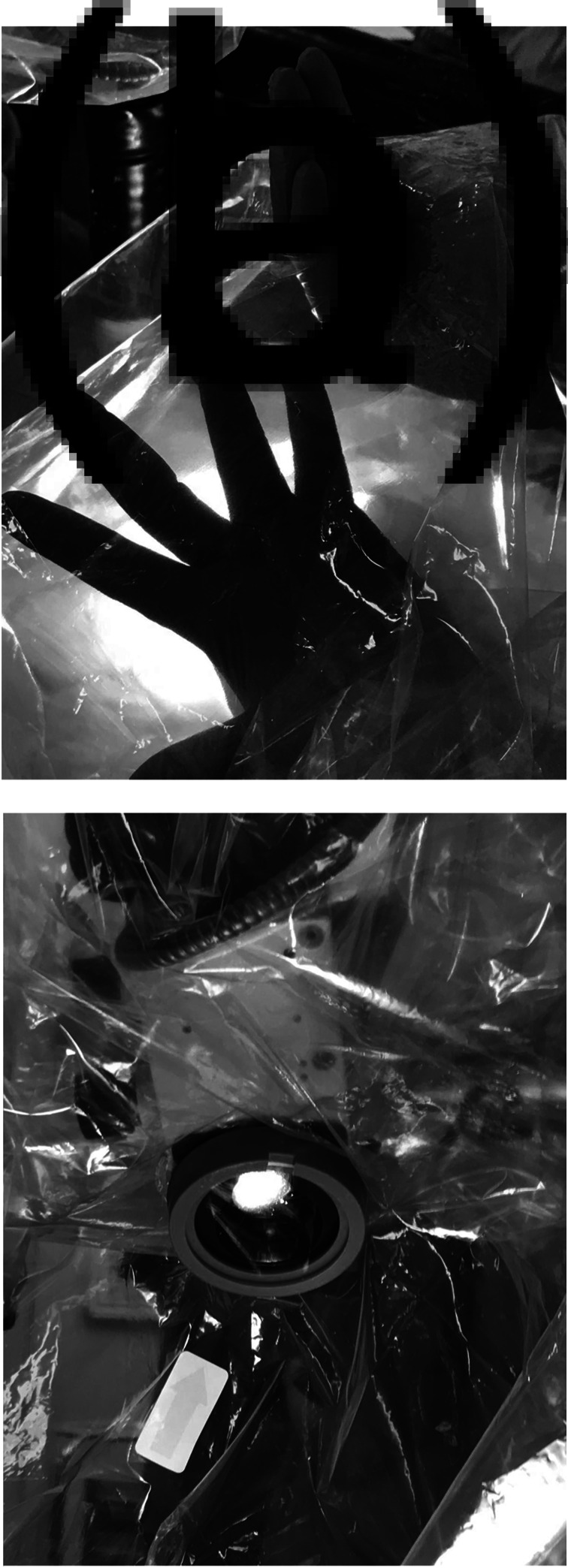
Attaching the novel drape ‘tent’ to the operating microscope. (a) Hole in the CE marked sterile polyethylene drape (intubation drape shield with endotracheal tube access (product reference: VED5010A); Vital Care Industries). (b) This hole is placed over the lens cap of the standard sterile microscope drape (Glass Lens Micro-Kover (product reference: 09-GL902); Advance Medical Designs) to create a secure fit.

**Fig. 2.**
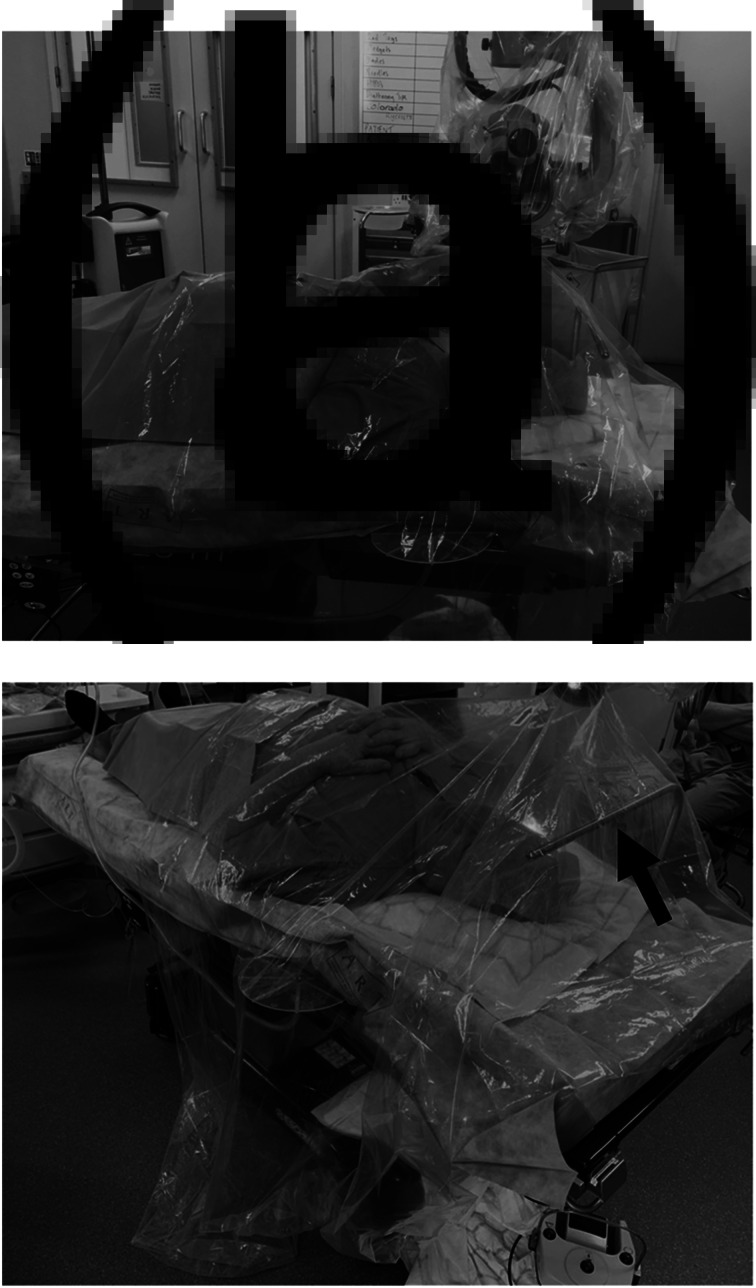
Example setup of the novel drape ‘tent’. The assembled drape ‘tent’ that was used for the paediatric patient has been recreated for illustrative purposes with the second author in place of the patient. (a) The CE marked sterile polyethylene drape (intubation drape shield with endotracheal tube access (product reference: VED5010A); Vital Care Industries) is attached to the lens cap of the standard sterile microscope drape (Glass Lens Micro-Kover (product reference: 09-GL902); Advance Medical Designs). The drape ‘tent’ is then extended to cover the entire surgical field. (b) The drape ‘tent’ is supported by an ‘L’ support (black arrow) at the head of the patient. During the mastoid surgery described in this case report, this ‘L’ support was covered in a sterile camera drape with ring applicator (product reference: 202-02; Delta Surgical).

The microscope (Opmi Vario/S88; Zeiss, Jena, Germany) was initially covered with a standard sterile microscope drape (Glass Lens Micro-Kover (product reference: 09-GL902); Advance Medical Designs, Marietta, Georgia, USA). The drape ‘tent’ used in this case consisted of a CE marked sterile polyethylene drape with a custom-made hole that fit securely over the lens cap of the standard microscope drape (intubation drape shield with endotracheal tube access (product reference: VED5010A); Vital Care Industries, Tinley Park, Illinois, USA (Figure 1)). The drape ‘tent’ was assembled by the operating surgeon and was placed over an ‘L’ support (covered in a sterile camera drape with ring applicator (product reference: 202-02); Delta Surgical, Newcastle under Lyme, UK) attached to the operating table at the patient's head. The drape was then extended to cover the entire patient and operating table to approximately the level of the patient's lower abdomen (Figure 2).

No modifications or alterations to the drape were required during assembly, and the visual field was unobscured by the drape (a hinderance of previous designs).^6,16–18^ In order to access the surgical field, the operating surgeon was required to pass their hands underneath the drape ‘tent’.

The time taken to assemble the drape ‘tent’ was 3 minutes and 30 seconds, and the time taken to disassemble the drape ‘tent’ was 2 minutes; hence, each process had a negligible effect on the total length of the surgical procedure.

The surgeon reported that the drape ‘tent’ did not affect their mobility, dexterity or tactility during the surgical procedure. No macroscopic droplet spread or particulate matter outside of the drape ‘tent’ was visualised by the surgeon at any point during the surgery.

## Discussion

The findings in this report align with evidence from pre-clinical studies, in which the use of a protective barrier in the form of a drape ‘tent’ substantially reduced the dispersion of particulate matter during temporal bone drilling.^16,17,19^

We report substantial advantages for this novel drape ‘tent’ over other designs. Specifically, the ‘Southampton Tent’, which involves the use of a second microscope drape to create a ‘tent’ over the patient, is reported to have a significant amount of ‘spare’ tent; this subsequently impacts on surgical efficiency in terms of instrument use.^6,18^ The ‘OtoTent’ utilises a 3M^™^ Steri-Drape^™^ 1060 that requires an incision to be made in the drape before it can be secured to the microscope lens mount.^16^ The Great Ormond Street Hospital drape ‘tent’ involves the customising and attaching of four overlapping panels of a 3M Steri-Drape 1060 to the side of a draped microscope.^18^ In contrast, the use of our novel drape ‘tent’ does not require any modifications for assembly, and no significant amount of ‘spare’ drape gathers around the surgical field. Furthermore, it is already CE marked for clinical use in its current form. We advocate the use of such a drape ‘tent’ during mastoid surgery in the Covid-19 era.
